# Electroencephalography Amplitude Modulation Analysis for Automated Affective Tagging of Music Video Clips

**DOI:** 10.3389/fncom.2017.00115

**Published:** 2018-01-10

**Authors:** Andrea Clerico, Abhishek Tiwari, Rishabh Gupta, Srinivasan Jayaraman, Tiago H. Falk

**Affiliations:** Centre Energie, Materiaux, Telecommunications, Institut National de la Recherche Scientifique, University of Quebec, Montreal, QC, Canada

**Keywords:** emotion classification, affective computing, multimedia content, electroencephalography, physiological signals, signal processing, pattern classification

## Abstract

The quantity of music content is rapidly increasing and automated affective tagging of music video clips can enable the development of intelligent retrieval, music recommendation, automatic playlist generators, and music browsing interfaces tuned to the users' current desires, preferences, or affective states. To achieve this goal, the field of affective computing has emerged, in particular the development of so-called affective brain-computer interfaces, which measure the user's affective state directly from measured brain waves using non-invasive tools, such as electroencephalography (EEG). Typically, conventional features extracted from the EEG signal have been used, such as frequency subband powers and/or inter-hemispheric power asymmetry indices. More recently, the coupling between EEG and peripheral physiological signals, such as the galvanic skin response (GSR), have also been proposed. Here, we show the importance of EEG amplitude modulations and propose several new features that measure the amplitude-amplitude cross-frequency coupling per EEG electrode, as well as linear and non-linear connections between multiple electrode pairs. When tested on a publicly available dataset of music video clips tagged with subjective affective ratings, support vector classifiers trained on the proposed features were shown to outperform those trained on conventional benchmark EEG features by as much as 6, 20, 8, and 7% for arousal, valence, dominance and liking, respectively. Moreover, fusion of the proposed features with EEG-GSR coupling features showed to be particularly useful for arousal (feature-level fusion) and liking (decision-level fusion) prediction. Together, these findings show the importance of the proposed features to characterize human affective states during music clip watching.

## 1. Introduction

With the rise of music and video-on-demand, as well as personalized recommendation systems, the need for accurate and reliable automated video tagging has emerged. In particular, user-centric affective tagging has stood out, corresponding to the formation of user emotional tags elicited while watching video clips (Kierkels et al., 2009; Shan et al., 2009; Koelstra and Patras, 2013). Emotions are usually conceived as physiological and physical responses, as part of natural communication between humans, and able to influence our intelligence, shape our thoughts and govern our interpersonal relationships (Marg, 1995; Loewenstein and Lerner, 2003; De Martino et al., 2006). Typically, machines were not required to have “emotion sensing” skills, but instead relied solely on interactivity. Recent findings from neuroscience, psychology and cognitive science, however, have modified this mentality and have pushed for such emotion sensing skills to be incorporated into machines. Such capability can allow machines to learn, in real-time, the user's preferences and emotions and adapt accordingly, thus taking the first steps toward the basic component of intelligence in human-human interaction (Preece et al., 1994).

Incorporating emotions into machines constitutes the burgeoning field of affective computing, which has as main purpose reduce the distance between the end-user and the machine by designing instruments that are able to accurately address human needs (Picard, 2000). To this end, the area of affective brain-computer interfaces (aBCIs) has recently emerged (Mühl et al., 2014). While BCIs have been mostly used to date for communication and rehabilitation applications (e.g., Li et al., 2006; Leeb et al., 2012; Sorensen and Kjaer, 2013), aBCIs (also known as passive BCIs) aim at measuring implicit information from the users, such as their moods and emotional states elicited by varying stimuli. Representative applications include neurogaming (Bos et al., 2010), neuromarketing (Lee et al., 2007), and “attention monitors” (Moore Jackson and Mappus, 2010), to name a few. As in Koelstra and Patras (2013), this paper concerns the measurement of emotions elicited on users by different music video clips, i.e., for automated multimedia tagging.

Within aBCIs, electroencephalography (EEG) has remained a popular modality due to its non-invasiveness, high temporal resolution (in the order of milliseconds), portability, and reasonable cost (Jenke et al., 2014). Typically, spectral features such as subband spectral powers have been used to measure emotional states elicited from music videos, pictures, and/or movie clips (e.g., Kierkels et al., 2009; Koelstra et al., 2012), as well as mental workload and stress (e.g., Heger et al., 2010; Kothe and Makeig, 2011). Moreover, an inter-hemispheric asymmetry in spectral power has been reported in the affective state literature (Davidson and Tomarken, 1989; Jenke et al., 2014), particularly in frontal brain regions (Coan and Allen, 2004).

Recent studies, however, have suggested that alternate EEG feature representations may exist that convey more discriminatory information over traditional spectral power and asymmetry indices (Jenke et al., 2014; Gupta and Falk, 2015). More specifically, statistical relations among temporal dynamics in different frequency bands (so-called “cross-frequency coupling”) have been observed in several brain regions and are thought to reflect neural communication and information encoding to support different perceptual and cognitive processes (Cohen, 2008) and emotional states (Schutter and Knyazev, 2012). Typically, cross-frequency coupling can be measured in three ways, namely, phase-phase, phase-amplitude and amplitude-amplitude coupling. While the former two have been widely studied and shown to be related to perception and memory (e.g., theta-gamma coupling Canolty et al., 2006), the latter has received lower attention. A few studies have shown amplitude-amplitude coupling effects on personality and motivation (Schutter and Knyazev, 2012) and recently, the authors proposed an inter-hemispheric cross-frequency amplitude coupling metric that correlated with affective states (Clerico et al., 2015). Notwithstanding, existing coupling metrics typically overlook temporal dynamics and are based on inter-hemispheric synchrony, thus overlook synchronization of other brain regions.

Moreover, in addition to EEG correlates, affective state information has been widely obtained from physiological signals measured from the peripheral autonomic nervous system (PANS) (Nasoz et al., 2003; Lisetti and Nasoz, 2004; Wu and Parsons, 2011), particularly the galvanic skin response (GSR), a measure of the amount of sweat (conductivity) in the skin (Picard and Healey, 1997; Bersak et al., 2001). More recently, the interaction between the PANS and central nervous systems (CNS) was measured via a phase-amplitude coupling (PAC) between GSR and EEG signals and promising emotion recognition results were found for highly arousing videos (Kroupi et al., 2014). As emphasized in Canolty et al. (2012), however, different ways of computing PAC may lead to complementary information. As such, in this paper we explore different PAC computation methods to gauge the advantages of one method over another.

In this paper, we build on the work of Clerico et al. (2015) and investigate the development of alternate features based on EEG amplitude modulation analysis for automated affective tagging of music video clips. In particular, we propose a number of innovations, namely: (1) extended the inter-hemispheric cross-frequency coupling measures of EEG amplitude modulations analysis to all possible electrode pairs, thus exploring connections beyond left-right pairs, (2) explored the use of a coherence based coupling metric, as opposed to mutual information, to explore linear relationships between inter-electrode coupling, (3) explored a total amplitude modulation energy measure to capture temporal dynamics, (4) proposed a normalization scheme based on normalization of the proposed features relative to a baseline period, thus facilitating cross-subject classification (as opposed to per-subject classification in Clerico et al., 2015), and (5) explored different ways of computing PAC between EEG and GSR in order to gauge the benefits of one computation method over another. Furthermore, we show the benefits of the proposed features relative to existing spectral power-based ones, and explore their complementarity via decision- and feature-level fusion. Experimental results show the proposed features outperforming conventional ones in recognizing arousal, valence, and dominance emotional primitives, as well as a “liking” subjective parameter.

The remainder of this paper is organized as follows: Section 2 provides the methodology used, including a description of the proposed and baseline features, as well as classification and fusion strategies used. Sections 3 and 4 describe the experimental results and discusses the findings, respectively. Lastly, section 5 presents the conclusions.

## 2. Materials and methods

In this section, the database, the proposed and benchmark feature sets, as well as the feature selection, classifier and classifier fusion schemes used are described.

### 2.1. Affective music clip audio-visual database

In this paper, the publicly-available DEAP (Dataset for Emotion Analysis using EEG and Physiological signals) database was used (Koelstra et al., 2012). Thirty-two healthy subjects (gender-balanced, average age of 26.9 years) were recruited to watch 40 video music clips while their neurophysiological signals were recorded. The forty videos were carefully selected from a larger set (roughly 200 videos), corresponding to the ones eliciting the 10 highest ratings within each of the four quadrants of the valence-arousal plane (Russell, 1980). Participants were asked to rate their perceived valence, arousal, and dominance emotional primitives, as well as other subjective ratings such as liking and familiarity for each of the 40 music clips. The three emotional primitives were scored using the 9-point continuous self-assessment manikin scale (Bradley and Lang, 1994). The liking scale was introduced to determine the user's taste, and not their feelings, about the music clip; as such, 9-point scale with thumbs down/up symbols was adopted. Lastly, the familiarity rating was scored using a 5-point scale. For the purpose of this paper, the familiarity rating was not used.

Several neurophysiological signals were recorded during music clip watching, namely 32-channel EEG (Biosemi Active II, with 10–20 international electrode placement), skin temperature, GSR, respiration, and blood volume pulse. The raw signals were recorded at a 512 Hz sample rate and down sampled offline to 128 Hz. The EEG signals were further bandpass filtered from 4 to 45 Hz, pre-processed using principal component analysis to remove ocular artifacts, averaged to a common reference and made publicly available. The interested reader is referred to Koelstra et al. (2012) for more details about the database.

### 2.2. Feature extraction

#### 2.2.1. Spectral features

**Figure 1:**
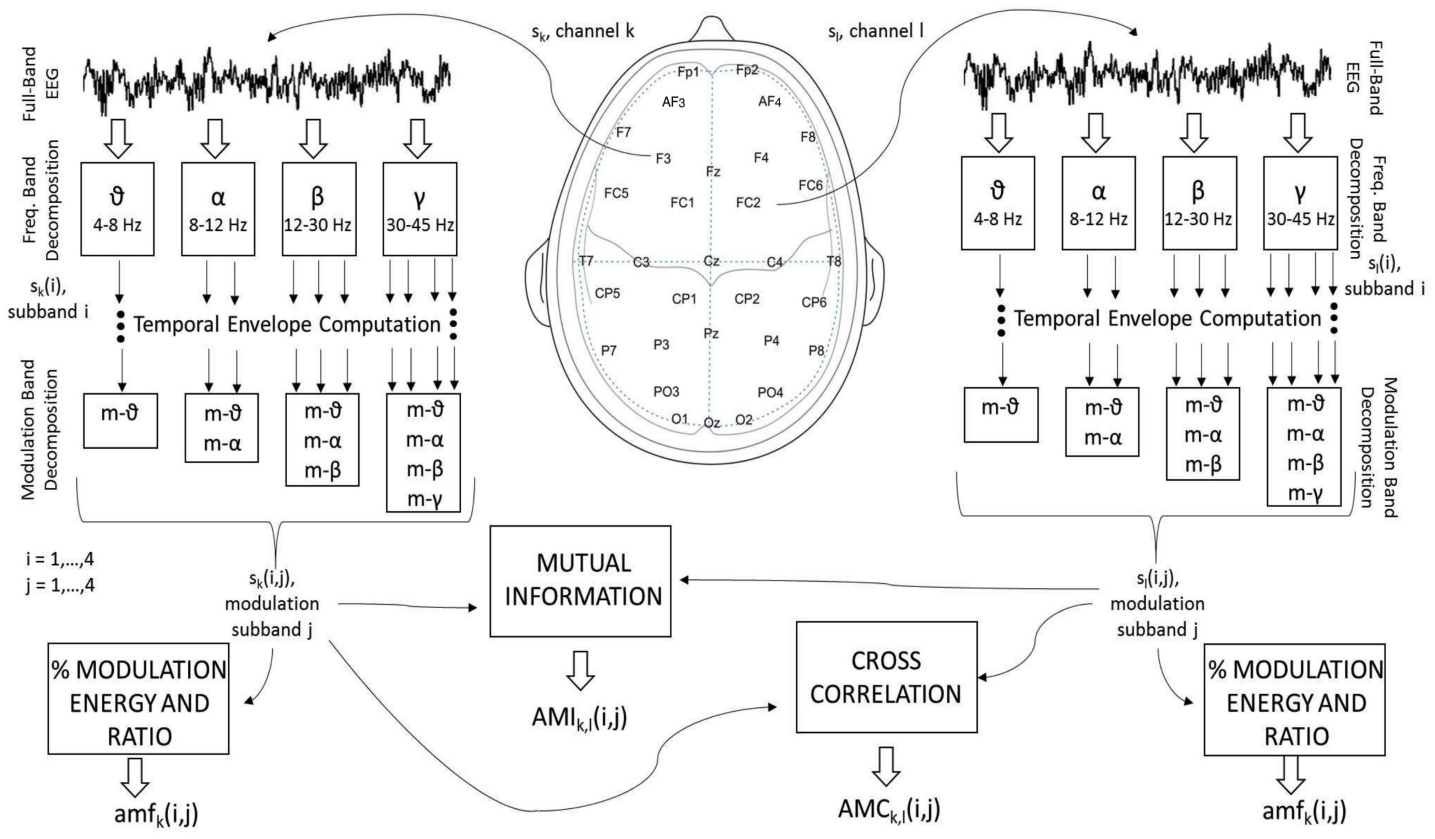
Signal processing steps used to compute the EEG amplitude modulation feature sets.

Spectrum subband power features are the most traditional measures used in biomedical signal processing (Sörnmo and Laguna, 2005). Within the affective state recognition literature, spectral power in the theta (4–8 Hz), alpha (8–12 Hz), beta (12–30 Hz), and gamma (30–45 Hz) subbands are typically used (Jenke et al., 2014) across different brain regions (Schutter et al., 2001; Balconi and Lucchiari, 2008). In particular, alpha and gamma band inter-hemispheric asymmetry indices have been shown to be correlated with emotional ratings, particularly in frontal brain regions (Müller et al., 1999; Mantini et al., 2007; Arndt et al., 2013). Given their widespread usage and the fact that they were also used in Koelstra et al. (2012) for affect recognition from the DEAP database, spectral features (“SF”) are used here as a benchmark to gauge the benefits of the proposed features. A total of 128 spectral power features (32 electrodes × 4 subbands) and 56 asymmetry indices (14 inter-hemispheric pairs × 4 subbands) were computed from the following electrode pairs: Fp1-Fp2, AF3-AF4, F7-F8, F3-F4, FC5-FC6, FC1-FC2, T7-T8, C3-C4, CP5-CP6, CP1-CP2, P7-P8, P3-P4, PO3-PO4, and O1-O2 (see Figure 1 for electrode labels and locations). Overall, a total of 184 “SF” features are used as benchmark.

#### 2.2.2. Amplitude modulation features

Cross-frequency amplitude-amplitude coupling in the EEG has been explored in the past as a measure of anxiety and motivation (e.g., Schutter and Knyazev, 2012), but has been under-explored within the affective state recognition community. Recently, beta-theta amplitude-amplitude coupling differences were observed between healthy elderly controls and age-matched Alzheimer's disease patients; such findings were linked to lack of interest and motivation within the patient population (Falk et al., 2012). To explore the benefits of cross-frequency amplitude-amplitude modulations for affective state recognition research, the authors recently showed that non-linear coupling patterns within inter-hemispheric electrode pairs was a reliable indicator of several affective dimensions, but particularly for the valence emotional primitive (Clerico et al., 2015). In this paper, we extend this work by extracting a number of other amplitude modulation features (“AMF”) and show their advantages for affective state recognition.

**Figure 2:**
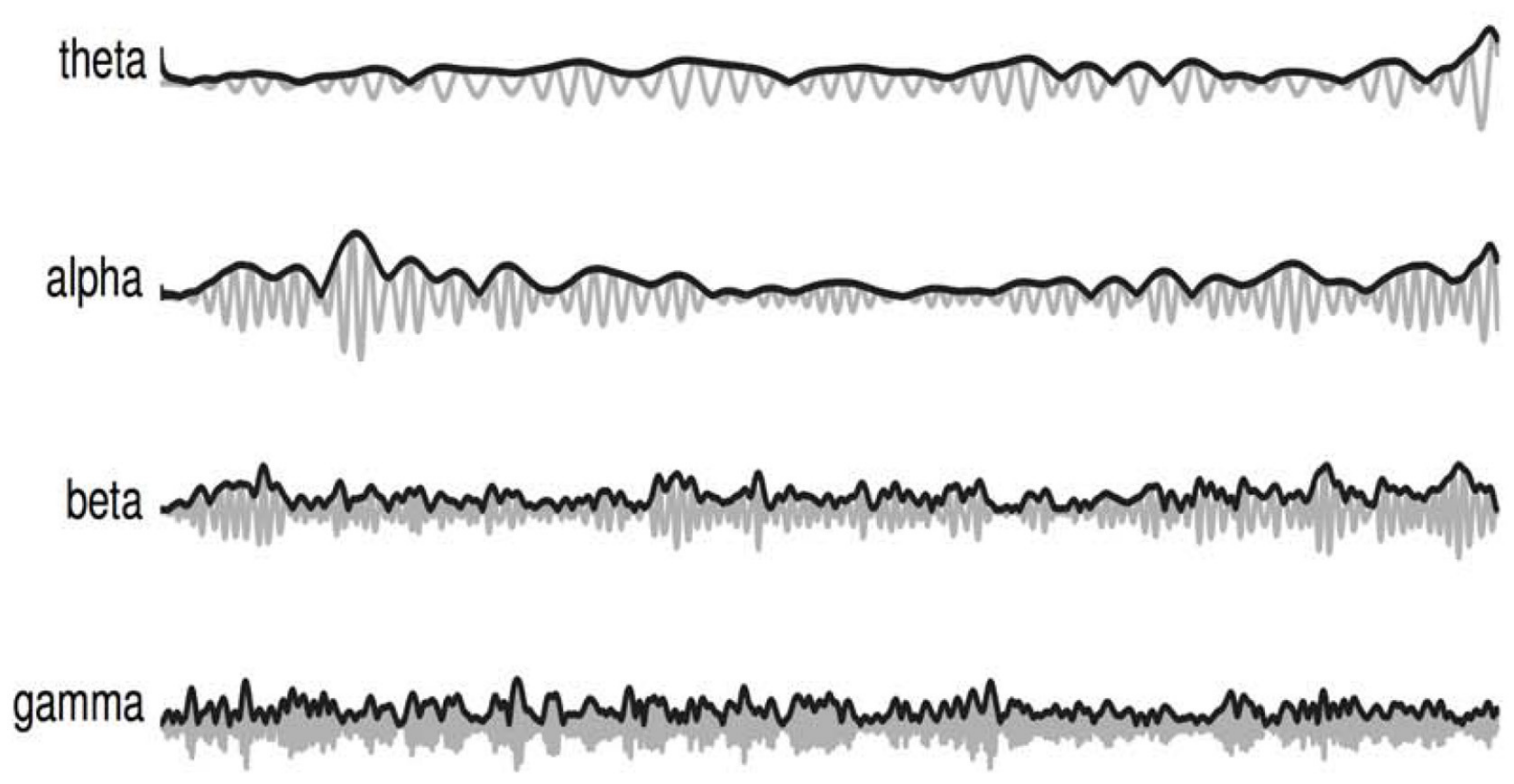
Amplitude envelope extraction from each EEG subband time series signal (gray) and their respective Hilbert amplitude envelopes (black).

More specifically, three new amplitude-amplitude coupling feature sets are extracted, namely the amplitude modulation energy (AME), amplitude modulation interaction (AMI), and the amplitude modulation coherence (AMC), as depicted by Figure 1. In order to compute these three feature sets, first the full-band EEG signal *s*_*k*_ for channel “k” (see left side of the figure) is decomposed into the four typical subbands (theta, alpha, beta and gamma) using zero-phase digital bandpass filters. Here, the time-domain index “n” is omitted for brevity, but without loss of generality. For the sake of notation, the decomposed time-domain signal is referred to as *s*_*k*_(*i*), *i* = 1, …, 4. The temporal envelope is then extracted from each of the four subband time series using the Hilbert transform (Le Van Quyen et al., 2001). Figure 2 illustrates the extracted EEG subband time series in gray and their respective Hilbert amplitude envelopes in black. Here, the temporal envelopes *e*_*i*_(*n*) of each subband time series were computed as the magnitude of the complex analytic signal $\zeta \left(n\right)=s_{k}{\left(i\right)}^{2}+jH\left\{s_{k}\left(i\right)\right\}$, i.e.,

(1) $$\begin{array}{l} e_{i}(i)=\sqrt{s_{k}{\left(i\right)}^{2}+H{\left\{s_{k}(i)\right\}}^{2}}, \end{array}$$

where, H{·} corresponds to the Hilbert transform.

In order to measure cross-frequency amplitude-amplitude coupling, a second decomposition of the EEG amplitude envelopes is performed utilizing the same four subbands. To distinguish between modulation and frequency subbands, the former are referred to as m-θ (4–8 Hz), m-α (8–12 Hz), m-β (12–30 Hz) and m-γ (30–45 Hz). For notation, the amplitude-amplitude coupling pattern is termed *s*_*k*_(*i, j*), *i, j* = 1, …, 4, where “i” indexes spectral subbands and “j” the modulation spectral subbands. By using the Hilbert transform to extract the amplitude envelope, the types of cross-frequency interactions are limited by Bedrosian's theorem, which states that the envelope signals can only contain frequencies (i.e., modulated frequencies) up to the maximum frequency of its original signal (Boashash, 1991; Smith et al., 2002). As such, only the ten cross-frequency patterns shown in Figure 1 are possible (per electrode), namely: θ_m-θ, α_m-θ, α_m-α, β_m-θ, β_m-α, β_m-β, γ_m-θ, γ_m-α, γ_m-β, and γ_m-γ. From these patterns, the three feature sets are computed, as detailed below:

##### 2.2.2.1. Amplitude modulation energy (AME)

From the ten possible *s*_*k*_(*i, j*) patterns per electrode, two energy measures are computed. The first measures the ratio of energy in a given frequency–modulation-frequency pair (ξ_*k*_(*i, j*)) over the total energy across all possible subbands pair (i.e., $\overset{4}{\underset{i=1}{\sum }}\overset{4}{\underset{j=1}{\sum }}\xi_{k}\left(i,j\right)$), thus resulting in 320 features (32 electrodes × 10 cross-frequency coupling patterns; see possible combinations in Figure 1). The second measures the logarithm of the ratio of modulation energy during the 60-s music clip to the modulation energy during a 3-s baseline resting period, i.e., $10\log\left(\xi_{k}{\left(i,j\right)}^{video}/\xi_{k}{\left(i,j\right)}^{baseline}\right)$, thus resulting in an additional 320 features, for a total of 640 AME_*k*_(*i, j*) features, *k* = 1, …, 32;*i, j* = 1, …, 4.

##### 2.2.2.2. Amplitude modulation interaction (AMI)

In order to incorporate inter-electrode amplitude modulation (non-linear) synchrony, the amplitude modulation interaction (AMI) features from Clerico et al. (2015) are also computed. Unlike the work described in Clerico et al. (2015), where interactions were only computed per symmetric inter-hemispheric pairs, here we measure interactions across all possible 496 electrode pair combinations (i.e., 2-by-2 combinations over all possible 32 channels) for each of the ten cross-frequency coupling patterns, thus resulting in 4960 features. The normalized mutual information (MI) is used to measure the interaction:

(2) $$\begin{array}{l} AMI_{k,l}=\frac{H(s_{k})+H(s_{l})-H(s_{k},s_{l})}{\sqrt{H(s_{k})H(s_{l})}}, \end{array}$$

where the *H*(·) operator represents marginal entropy and *H*(·, ×) the joint entropy, and *s*_*k*_ corresponds to *s*_*k*_(*i, j*) with the frequency and modulation frequency indices omitted for brevity. Entropy was calculated using the histogram method with 50 discrete bins for each variable. Mutual information has been used widely in affective recognition research (e.g., Cohen et al., 2003; Khushaba et al., 2012; Hamm et al., 2014). Additionally a second measurement of logarithmic ratio between the 60-s clip and the 3-s baseline has been obtained, thus totalling 9920 AMI features.

##### 2.2.2.3. Amplitude modulation coherence (AMC)

While the AMI features capture non-linear interactions between inter-electrode amplitude-amplitude coupling patterns, the Pearson correlation coefficient between the patterns can also be used to quantify the coherence, or linear interactions between the patterns. Spectral coherence measures have been widely used in EEG research and were recently shown to also be useful for affective state research (e.g., Kar et al., 2014; Xielifuguli et al., 2014). Hence, we explore the concept of amplitude modulation coherence, or AMC as a new feature for affective state recognition. The AMC features are computed as:

(3) $$\begin{array}{l} AMC_{k,l}=\frac{\sum_{n=1}^{N}\left(s_{k}(n)-{\bar{s}}_{k})(s_{l}(n)-{\bar{s}}_{l}\right)}{\sqrt{\sum_{n=1}^{N}(s_{k}n)-{\bar{s}}_{k}{)}^{2}\sum_{n=1}^{N}{\left(s_{l}(n)-{\bar{s}}_{1}\right)}^{2}}}, \end{array}$$

where *s*_*k*_(*n*) indicates the n-th sample of the *s*_*k*_(*i, j*) time-series (again, the frequency and modulation frequency indices were omitted for brevity), and $\bar{s_{k}}$ is the average over all samples of such time series. As previously, a total of 9920 AMC features are computed, including the logarithmic ratio with the 3-s baseline.

#### 2.2.3. PANS-CNS phase-amplitude coupling (PAC)

Electrophysiological signals reflect dynamical systems that interact with each other at different frequencies. Phase-Amplitude coupling represents one type of interaction and typically refers to modulation of the amplitude of high-frequency oscillators by the phase of low-frequency ones (Samiee et al.). Typically, such phase-amplitude coupling measures are computed from EEG signals alone (Schutter and Knyazev, 2012), but the concept of electrodermal activity phase coupled to EEG amplitude was recently introduced as a correlate of emotion, particularly for high arousing, very pleasant and very unpleasant stimuli (Kroupi et al., 2013, 2014). Here, we test three different GSR-phase and EEG-amplitude coupling measures. For the sake of notation, assume *u*(*n*) is the rapid transient response called skin conductance response (SCR) with a narrowband of 0.5–1Hz (Kroupi et al., 2014), of the time-domain GSR signal. Using the Hilbert transform (Gabor, 1946), we can extract the signal's instantaneous phase ϕ_*u*_(*n*) as in Kroupi et al. (2014):

(4) $$\begin{array}{l} \phi_{u}\left(n\right)=arctan\left(\frac{H\{u(n)\}}{u\left(n\right)}\right). \end{array}$$

For the amplitude envelope of the EEG signal (*A*(*s*_*k*_(*n*))), a shape-preserving piecewise cubic interpolation method of neighboring values is used, as in Kroupi et al. (2014). Given the GSR signal and phase, as well as the EEG amplitude envelope signals, the following coupling measures were computed.

##### 2.2.3.1. Envelope-to-signal coupling (ESC)

The simplest coupling feature can be calculated via the Pearson correlation coefficient between the EEG amplitude envelope signal *A*(*s*_*k*_(*n*)) and the raw GSR signal *u*(*n*). The ESC feature can be computed using equation (4) with *A*(*s*_*k*_(*n*)) and *u*(*n*) in lieu of *s*_*k*_(*i, j*) and *s*_*l*_(*i, j*), respectively (Arnulfo et al., 2015). ESC has been shown to be particularly useful with noisy data (Onslow et al., 2011). A total of 32 ESC features were computed.

##### 2.2.3.2. Cross-frequency coherence (CFC)

Cross-frequency coherence evaluates the magnitude square coherence between the filtered (0-1 Hz) GSR signal *u*(*n*) and the filtered (4–45 Hz) envelope of the EEG signal *A*(*s*_*k*_(*n*)), as in Onslow et al. (2011). The CFC feature is computed as:

(5) $$\begin{array}{l} CFC_{k}(f)=\frac{|P_{Au}(f){|}^{2}}{P_{AA}(f)P_{uu}(f)}, \end{array}$$

where $|P_{Au}\left(f\right){|}^{2}$ is the cross power spectral density of the EEG amplitude *A*(*s*_*k*_(*n*)) and GSR signal *u*(*n*) at frequency *f*, and *P*_*AA*_(*f*) and *P*_*uu*_(*f*) are the spectral power densities of the two signals, respectively. The CFC feature ranges from 0 (no spectral coherence) to 1 (perfect spectral coherence) and has been used previously to quantify linear EEG synchrony in different frequency bands and its relationship with emotions (Daly et al., 2014). A total of 1344 CFC features were computed.

##### 2.2.3.3. Modulation index (ModI)

PANS-CNS coupling measure tested is the so-called modulation index (ModI), which was recently shown to accurately characterize coupling intensity (Tort et al., 2010), particularly for emotion recognition (Kroupi et al., 2014). For calculation of the ModI feature, a composite times series is constructed as [ϕ_*u*_(*n*), *A*(*s*_*k*_(*n*))]. The phases are then binned and the mean of *A*(*s*_*k*_(*n*)) over each phase bin is calculated and denoted by 〈*A*_*s*_〉ϕ_*u*_(*m*), where m indexes phase bin; 18 bins were used in this experiment. Further, the mean amplitude distribution *P*(*m*) is normalized by the sum over all bins, i.e.,:

(6) $$\begin{array}{l} P(m)=\frac{\langle A_{s}\rangle \phi_{u}\left(m\right)}{\sum_{m=1}^{18}\langle A_{s}\rangle \phi_{u}\left(m\right)}. \end{array}$$

The normalized amplitude “distribution” *P*(*m*) has similar properties as a probability density function. In fact, in the scenario in which no phase-amplitude coupling exists, *P*(*n*) assumes a uniform distribution. Having this said, the ModI feature measures the deviation of *P*(*m*) from a uniform distribution. This is achieved by means of a Kullback-Liebler (KL) divergence measure (Kullback and Leibler, 1951) between *P*(*m*) and a uniform distribution *Q*(*m*), given by:

(7) $$\begin{array}{l} D_{KL}\left(P,Q\right)=\sum_{m=1}^{18}U\left(m\right)log\left[\frac{P\left(m\right)}{Q\left(m\right)}\right], \end{array}$$

The KL divergence *D*_*KL*_(*P, Q*) is always greater than zero, and equal to zero only when the two distributions are the same. Finally, the ModI feature is defined as the ratio between the KL divergence and the log of the number of phase bins, i.e.,:

(8) $$\begin{array}{l} ModI=\frac{D_{KL}\left(P,Q\right)}{log\left(M\right)}. \end{array}$$

where *M* = 18 is used in our experiments. A total of 32 ModI features were computed.

### 2.3. Feature selection and affective state recognition

In this section, a description of the feature selection, classifiers, and classifier fusion strategies are discussed.

#### 2.3.1. Feature selection

As mentioned above, a large number of proposed and benchmark features were extracted. More specifically, a total of 184 SF, 20480 AMF, and 1408 PAC features were extracted. For classification purposes, these numbers are large and may lead to classifier overfitting. In such instances, feature ranking and/or feature selection algorithms are typically used. Recently, several feature selection algorithms were compared on an emotion recognition task (Jenke et al., 2014). The minimum redundancy maximum relevance (mRMR) algorithm (Peng et al., 2005) showed improved performance when paired with a support vector machine classifier (Wang et al., 2011). The mRMR is a mutual information based algorithm that optimizes two criteria simultaneously: the maximum-relevance criterion (i.e., maximizes the average mutual information between each feature and the target vector) and the minimum-redundancy criterion (i.e., minimizes the average mutual information between two chosen features). The algorithm finds near-optimal features using forward selection with the chosen features maximizing the combined max-min criteria.

Moreover, in an allied domain, multi-stage feature selection comprised of analysis of variance (ANOVA) between the features and target labels as a pre-screening, followed by mRMR, was shown to lead to improved results for SVM-based classifiers (Dastgheib et al., 2016). This multi-stage feature selection procedure is explored herein and during pre-screening, only features that attained *p*-values smaller than 0.1 were kept. Here, two tests are explored. With one, all top selected features for each feature class are used for classifier training. Given the different number of available features for each feature class, the input dimensionality of the attained classifiers will differ. For a more fair comparison, the second assumes that classifiers are trained on the same number of features for each feature class. To this end, the number of features used corresponds to the number of benchmark SF features that pass the ANOVA test.

In the available dataset, neurophysiological signals were recorded from 32 subjects while each watched a total of 40 music clips. Here, 25% of the available data (i.e., data from 10 music clips per subject, roughly half from the high and half from the low classes) was set aside for feature ranking. The remaining 75% was used for classifier training and testing in a leave-one-sample-out (LOSO) cross-validation scheme, as described next. This hold-out scheme assures a more stringent setup, as feature selection and model training are not performed on the same data subset, which could lead to overly optimistic results. From the feature selection set, it was found that 35, 23, 19, and 21 SF features passed the ANOVA test for arousal, valence, dominance, and liking dimensions, respectively.

#### 2.3.2. Classification

During pilot phase, support vector machine (SVM), relevance vector machine (RVM) and random forest classifiers were explored. Overall, SVMs resulted in improved performance. Indeed, they have been widely used in bioengineering and in affective state recognition (e.g., Wang et al., 2011). Given their widespread use, a description of the support vector machine approach is not included here and the interested reader is referred to Schölkopf and Smola (2002) and references therein for more details. Here, SVM classifiers are trained on four different binary classification problems, i.e., detecting low/high valence, low/high arousal, low/high dominance and low/high liking.

**Figure 3:**
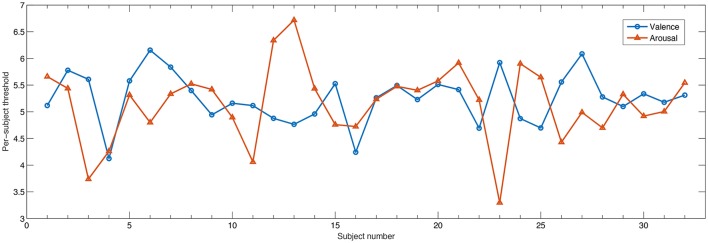
Individualized threshold such that approximately 50/50 ratio was achieved for high/low class for valence and arousal dimensions.

With the DEAP database, subjective ratings followed a 9-point scale. Typically, values greater or equal to 5 are assumed to correspond to high activation levels or low, otherwise. However, it is not guaranteed that all users objectively utilize the same scale for grading. In fact, by using a threshold of 5, a 60/40 ratio of high/low levels was obtained across all participants. In order to take into account individual biases during rating, here we utilize an individualized threshold corresponding to the value in which an almost balanced high/low ratio was achieved per participant. Figure 3 depicts the threshold found for each participant for arousal and valence. As can be seen, on average a threshold of 5 was most often selected, though in a few cases, much higher or much lower values were found, thus exemplifying the need for such an individualized approach.

As mentioned previously, 75% of the available dataset was used for classifier training/testing using a leave-one-sample-out (LOSO) cross-validation scheme. For our experiments, a radial basis function (RBF) kernel was used and implemented with the Scikit-learn library in Python (Pedregosa et al., 2011). Since we are interested in gauging the benefits of the proposed features, and not of the classification schemes, we use the default SVM parameters throughout our experiments (i.e., λ = 1 and γ_*RBF*_ = 0.01). As such, it is expected that improved performance should be achieved once classifier optimization is performed, as in Gupta et al. (2016). Such analysis, however, is left for future study.

#### 2.3.3. Fusion

In an attempt to improve classification performance, two fusion strategies are explored, namely, feature fusion and decision-level fusion. In feature fusion, we explore the combination of the three feature sets (SF, PAC, and AMF) and utilize the top selected features. With classifier decision-level fusion, on the other hand, the decisions of the three SVM classifiers trained on the top SF, PAC, and AMF sets were fused using a simple majority voting scheme with equal weights.

### 2.4. Figure of merit

Balanced accuracy (BACC) is used as a figure of merit and corresponds to the arithmetic mean of the classifier sensitivity and specificity, namely:

(9) $$\begin{array}{l} BACC=\frac{SENS+SPEC}{2}, \end{array}$$

where

(10) $$\begin{array}{l} SENS=\frac{TP}{P};SPEC=\frac{TN}{N}, \end{array}$$

and *P* = *TP* + *FN* and *N* = *FP* + *TN*, TP and FP correspond to true and false positives, respectively and TN and FN to true and false negatives, respectively. Balanced accuracy takes into account any remaining class unbalances and provides more accurate results than the conventional accuracy metric. To test the significance of the attained performances, an independent one-sample *t*-test against a random voting classifier was used (*p* < 0.05), as suggested in Koelstra et al. (2012).

## 3. Results

Tables 1–4 show the top-selected features for the arousal, valence, dominance, and liking dimensions, respectively, following multi-stage feature selection and using the same number of features across sets. Feature names listed in the tables should be self explanatory. The “ratio” features correspond to the log-ratio ones between the video and baseline periods (see section 2.2.2). In the SF category, the “AI” features correspond to the asymmetry index between the indicated channels.

**Table 1:** Selected top-35 features for the arousal dimension.

**Ranking**	**Arousal**				
	**AMI**	**AMC**	**AME**	**PAC**	**SF**
1	γ_m-γ_FC5_CP2	α_m-α_T8_CP6	ratio_γ_m-γ_Fz	cfc_FC1_7_Hz	AI_β_FC1_FC2
2	β_m-α_FC5_CP5	θ_m-θ_Fp1_Pz	β_m-α_F7	cfc_CP5_7_Hz	AI_θ_FC1_FC2
3	γ_m-γ_FC5_Cz	γ_m-γ_FC5_FC1	ratio_γ_m-β_Pz	cfc_O1_19_Hz	γ_Fp1
4	γ_m-γ_FC5_AF4	γ_m-θ_CP5_F8	θ_m-θ_O1	cfc_FC5_15_Hz	θ_O2
5	γ_m-γ_AF4_CP2	θ_m-θ_C3_O2	ratio_β_m-θ_CP5	cfc_FC1_44_Hz	α_O2
6	β_m-α_CP5_Pz	α_m-α_P7_C4	β_m-β_O2	cfc_O1_20_Hz	α_F7
7	γ_m-γ_FC5_PO4	α_m-θ_F7_T7	ratio_α_m-θ_O2	cfc_O1_27_Hz	θ_CP6
8	γ_m-α_PO3_F8	γ_m-γ_P7_F8	γ_m-β_F7	cfc_O1_28_Hz	α_Pz
9	γ_m-γ_FC5_C4	β_m-θ_C4_P4	ratio_α_m-α_T8	cfc_FC5_16_Hz	AI_β_AF3_AF4
10	γ_m-β_FC5_PO4	θ_m-θ_FC6_Cz	ratio_β_m-β_FC2	cfc_FC1_39_Hz	β_FC5
11	β_m-θ_FC2_P8	α_m-θ_T8_CP6	θ_m-θ_FC5	cfc_FC1_43_Hz	θ_AF4
12	γ_m-γ_FC5_Fp2	θ_m-θ_Fp1_P7	ratio_α_m-θ_Cz	cfc_FC1_42_Hz	θ_P4
13	γ_m-γ_FC5_Fz	γ_m-θ_P7_F8	ratio_β_m-θ_Pz	cfc_O1_18_Hz	AI_β_P7_P8
14	γ_m-γ_AF4_Cz	α_m-α_FC2_P8	α_m-α_Cz	cfc_O1_26_Hz	θ_F8
15	β_m-β_AF3_CP5	α_m-θ_P7_C4	ratio_α_m-α_O2	cfc_P8_5_Hz	AI_β_FC5_FC6
16	β_m-β_FC5_CP5	β_m-α_C4_P4	ratio_α_m-θ_Fz	cfc_FC1_37_Hz	β_Fp2
17	α_m-α_FC1_T8	θ_m-θ_C3_O1	ratio_α_m-α_Cz	cfc_O1_29_Hz	θ_FC6
18	α_m-α_Oz_CP2	θ_m-θ_P3_P8	γ_m-α_F7	cfc_O1_23_Hz	θ_T8
19	γ_m-γ_FC5_FC6	α_m-θ_Fp1_Cz	α_m-θ_O2	cfc_O1_22_Hz	α_Fz
20	β_m-β_PO3_P8	γ_m-α_T7_FC2	ratio_β_m-θ_P3	cfc_FC1_8_Hz	α_PO3
21	γ_m-β_AF4_PO4	γ_m-β_FC5_FC1	α_m-θ_T8	cfc_FC5_18_Hz	γ_F4
22	γ_m-β_FC5_Fz	β_m-θ_T7_T8	ratio_γ_m-γ_Oz	cfc_FC1_35_Hz	AI_θ_O1_O2
23	β_m-α_AF3_Pz	γ_m-α_FC5_FC1	θ_m-θ_P7	cfc_Fz_19_Hz	θ_P8
24	γ_m-γ_AF4_PO4	β_m-θ_Cz_PO4	ratio_γ_m-θ_CP1	esc_C4	AI_β_Fp1_Fp2
25	γ_m-β_FC5_Fp2	θ_m-θ_O1_CP6	α_m-θ_CP6	cfc_CP1_5_Hz	β_F3
26	γ_m-γ_Fp2_AF4	γ_m-γ_CP5_F8	α_m-α_T8	cfc_O1_25_Hz	β_FC1
27	α_m-θ_PO3_CP2	γ_m-γ_T7_FC2	β_m-α_C3	cfc_FC1_41_Hz	γ_P3
28	γ_m-γ_FC5_P3	β_m-β_F3_PO3	ratio_γ_m-γ_Pz	cfc_FC1_40_Hz	β_Fp1
29	γ_m-γ_FC5_FC1	γ_m-β_T7_FC2	ratio_γ_m-θ_Pz	cfc_FC1_38_Hz	α_PO4
30	β_m-β_AF3_O2	β_m-β_C4_P4	ratio_α_m-α_Fz	cfc_O1_30_Hz	θ_Fp2
31	α_m-θ_FC1_T8	α_m-θ_FC2_P8	ratio_γ_m-θ_P3	cfc_FC5_20_Hz	α_F4
32	α_m-θ_F3_Oz	γ_m-β_CP5_F8	α_m-θ_Cz	cfc_FC5_19_Hz	α_P7
33	γ_m-β_FC5_FC6	γ_m-β_P7_F8	ratio_θ_m-θ_O2	cfc_O1_21_Hz	AI_β_F7_F8
34	γ_m-γ_F3_Fp2	α_m-α_F7_T7	β_m-β_F7	cfc_FC5_17_Hz	β_AF3
35	γ_m-γ_FC1_AF4	α_m-α_Fp1_Cz	ratio_α_m-θ_AF4	cfc_O1_24_Hz	θ_CP2

*Feature names listed should be self explanatory. The “ratio” features correspond to the log-ratio ones between the video and baseline periods; “AI” corresponds to the asymmetry index between the indicated channels*.

**Table 2:** Selected top-23 features for the valence dimension.

**Ranking**	**Valence**				
	**AMI**	**AMC**	**AME**	**PAC**	**SF**
1	α_m-α_O1_CP2	θ_m-θ_T7_F8	β_m-θ_PO4	cfc_T8_5_Hz	AI_α_PO3_PO4
2	α_m-α_O1_Oz	β_m-β_AF3_F4	ratio_γ_m-α_PO3	cfc_C3_26_Hz	α_P7
3	α_m-θ_F7_Pz	γ_m-γ_CP1_P7	ratio_β_m-β_Fp1	cfc_CP1_25_Hz	γ_Fz
4	α_m-α_F3_O1	γ_m-θ_AF3_Oz	ratio_α_m-θ_Oz	cfc_CP1_28_Hz	α_P3
5	α_m-α_O1_Fp2	β_m-α_F7_P8	ratio_γ_m-β_PO3	cfc_O2_15_Hz	AI_α_P3_P4
6	α_m-α_O1_O2	γ_m-β_F3_Oz	β_m-θ_Pz	cfc_C3_25_Hz	AI_γ_O1_O2
7	α_m-α_T7_O1	γ_m-γ_AF3_P7	ratio_γ_m-β_Fp1	cfc_C3_24_Hz	θ_Fz
8	β_m-β_CP6_CP2	γ_m-θ_AF3_P7	ratio_β_m-α_Fp1	esc_F3	α_PO3
9	α_m-θ_O1_CP2	γ_m-α_F3_Oz	γ_m-β_PO4	cfc_O2_14_Hz	AI_α_P7_P8
10	β_m-β_F4_CP2	θ_m-θ_Pz_PO4	ratio_β_m-θ_P8	cfc_FC1_42_Hz	θ_O1
11	β_m-θ_AF3_Oz	α_m-α_Fp1_Pz	β_m-β_P3	cfc_FC1_43_Hz	β_PO3
12	α_m-α_O1_PO4	θ_m-θ_F4_FC2	ratio_α_m-α_CP2	cfc_C3_27_Hz	AI_α_F7_F8
13	β_m-β_F4_F8	γ_m-γ_F3_Oz	γ_m-γ_PO4	cfc_CP1_23_Hz	AI_γ_C3_C4
14	γ_m-β_F7_Cz	γ_m-θ_F3_Oz	β_m-θ_T8	cfc_C3_23_Hz	AI_γ_FC1_FC2
15	α_m-θ_O1_O2	β_m-α_AF3_F4	β_m-α_P3	cfc_CP1_30_Hz	AI_β_PO3_PO4
16	α_m-θ_O1_Cz	γ_m-θ_Oz_O2	β_m-α_PO4	cfc_CP1_24_Hz	AI_β_FC5_FC6
17	α_m-α_CP1_PO4	β_m-α_F4_P8	β_m-β_T7	esc_F4	AI_α_O1_O2
18	γ_m-θ_F3_O1	γ_m-α_CP1_P7	β_m-θ_T7	cfc_FC1_45_Hz	AI_β_F7_F8
19	γ_m-β_P8_O2	γ_m-β_CP1_P7	ratio_β_m-α_PO3	cfc_CP1_26_Hz	AI_θ_AF3_AF4
20	α_m-θ_O1_Fz	β_m-β_CP5_T8	γ_m-θ_PO4	cfc_CP1_29_Hz	α_Fz
21	α_m-θ_F7_AF4	β_m-β_F7_P8	ratio_β_m-β_PO3	cfc_CP1_27_Hz	AI_β_P3_P4
22	α_m-α_O1_Cz	γ_m-β_AF3_P7	ratio_θ_m-θ_CP2	cfc_FC1_44_Hz	β_P3
23	α_m-θ_O1_Oz	θ_m-θ_O1_Cz	ratio_γ_m-α_Fp1	esc_AF3	θ_AF3

*Feature names listed should be self explanatory. The “ratio” features correspond to the log-ratio ones between the video and baseline periods; “AI” corresponds to the asymmetry index between the indicated channels*.

**Table 3:** Selected top-19 features for the dominance dimension.

**Ranking**	**Dominance**				
	**AMI**	**AMC**	**AME**	**PAC**	**SF**
1	θ_m-θ_CP1_T8	β_m-θ_P7_F8	γ_m-β_P7	esc_AF3	θ_FC2
2	α_m-α_P3_Oz	β_m-α_CP1_F8	β_m-β_P3	cfc_FC2_11_Hz	γ_F3
3	α_m-θ_AF3_T7	β_m-θ_T7_F8	α_m-α_Pz	cfc_FC2_8_Hz	α_PO3
4	γ_m-α_F7_CP6	β_m-α_CP5_AF4	γ_m-α_P7	cfc_CP6_7_Hz	θ_C3
5	θ_m-θ_P3_P8	β_m-β_CP1_Fz	γ_m-θ_PO4	cfc_CP6_8_Hz	θ_Pz
6	θ_m-θ_FC2_P8	β_m-α_P7_FC6	α_m-θ_Pz	cfc_F3_6_Hz	γ_P7
7	β_m-α_CP1_Pz	β_m-θ_PO3_P4	ratio_γ_m-β_P8	cfc_FC2_7_Hz	θ_FC6
8	α_m-α_F3_Fz	β_m-α_CP1_Fz	ratio_β_m-θ_P8	cfc_FC5_5_Hz	θ_P4
9	β_m-θ_P3_F4	β_m-θ_F8_P4	ratio_γ_m-γ_P8	cfc_F4_12_Hz	β_F3
10	β_m-α_CP1_P3	β_m-β_CP1_F8	θ_m-θ_Pz	cfc_FC2_9_Hz	α_P7
11	β_m-α_P3_Pz	β_m-α_PO3_PO4	γ_m-γ_PO4	cfc_FC5_11_Hz	β_C4
12	β_m-θ_P3_PO4	β_m-α_P7_F8	ratio_γ_m-γ_PO4	cfc_CP1_42_Hz	AI_β_CP5_CP6
13	α_m-α_AF3_Fz	β_m-β_CP5_Pz	β_m-α_F7	cfc_CP6_9_Hz	θ_PO3
14	β_m-β_FC5_Pz	β_m-α_CP5_Pz	γ_m-β_F7	cfc_P4_6_Hz	α_Pz
15	α_m-α_AF3_T7	β_m-β_CP5_AF4	γ_m-β_P3	cfc_F4_14_Hz	θ_P3
16	γ_m-α_F7_CP2	β_m-β_P7_F8	γ_m-γ_F7	cfc_AF4_5_Hz	θ_Fp2
17	θ_m-θ_CP1_CP2	β_m-β_PO3_PO4	γ_m-θ_Cz	cfc_F4_13_Hz	AI_β_PO3_PO4
18	θ_m-θ_T8_P8	β_m-θ_CP1_F8	β_m-β_Cz	cfc_FC2_12_Hz	θ_O1
19	β_m-β_FC5_P3	β_m-θ_CP1_Fz	β_m-β_F7	cfc_FC2_10_Hz	AI_γ_F7_F8

*Feature names listed should be self explanatory. The “ratio” features correspond to the log-ratio ones between the video and baseline periods; “AI” corresponds to the asymmetry index between the indicated channels*.

**Table 4:** Selected top-21 features for the liking dimension.

**Ranking**	**Liking**				
	**AMI**	**AMC**	**AME**	**PAC**	**SF**
1	β_m-θ_AF4_CP6	γ_m-α_Pz_AF4	ratio_β_m-θ_FC6	cfc_P7_30_Hz	α_P3
2	β_m-α_PO3_P8	β_m-β_O1_T8	γ_m-β_P7	cfc_FC1_7_Hz	θ_C3
3	α_m-α_O1_Oz	β_m-β_Pz_FC2	ratio_γ_m-γ_P8	cfc_P7_29_Hz	β_P3
4	γ_m-γ_Fp1_T7	γ_m-β_Pz_AF4	γ_m-θ_P3	cfc_PO4_42_Hz	β_T8
5	α_m-α_Oz_FC2	β_m-β_CP5_AF4	α_m-α_AF4	esc_T8	β_O1
6	θ_m-θ_Fp1_AF4	γ_m-γ_Pz_AF4	ratio_γ_m-α_P8	cfc_P7_32_Hz	θ_P4
7	θ_m-θ_C3_P8	β_m-θ_FC1_O1	ratio_γ_m-θ_F3	cfc_P7_31_Hz	α_F8
8	θ_m-θ_CP5_AF4	γ_m-θ_Pz_AF4	α_m-α_CP1	cfc_PO4_39_Hz	β_PO3
9	β_m-θ_P3_AF4	γ_m-γ_AF3_Oz	ratio_β_m-θ_P8	cfc_PO4_45_Hz	AI_β_FC5_FC6
10	β_m-θ_F7_AF4	γ_m-β_CP1_AF4	γ_m-θ_F3	esc_F3	β_AF3
11	θ_m-θ_P7_AF4	β_m-α_O1_T8	ratio_β_m-β_C3	cfc_PO4_44_Hz	θ_CP1
12	β_m-β_PO3_P8	γ_m-α_Fp1_T7	ratio_β_m-α_C3	cfc_Fp1_8_Hz	α_CP5
13	θ_m-θ_PO3_Cz	β_m-α_CP5_P4	ratio_β_m-α_F3	cfc_P7_26_Hz	β_F3
14	β_m-θ_F3_AF4	γ_m-θ_CP1_AF4	ratio_α_m-θ_Fp1	esc_CP1	AI_α_P7_P8
15	θ_m-θ_CP1_AF4	γ_m-β_AF3_Oz	ratio_γ_m-γ_FC6	cfc_PO4_41_Hz	AI_β_F7_F8
16	α_m-θ_Oz_FC2	β_m-θ_FC5_PO3	ratio_β_m-α_Fp2	cfc_PO4_43_Hz	AI_β_PO3_PO4
17	α_m-θ_P7_P8	γ_m-γ_CP1_AF4	ratio_γ_m-β_P8	cfc_P7_27_Hz	θ_FC6
18	β_m-β_PO3_P4	γ_m-α_AF3_Oz	β_m-θ_P7	esc_P8	β_FC5
19	θ_m-θ_Pz_CP6	β_m-θ_F3_P8	ratio_θ_m-θ_P7	cfc_FC1_8_Hz	AI_θ_F7_F8
20	θ_m-θ_Pz_AF4	β_m-β_F3_P8	ratio_β_m-β_F3	cfc_FC6_10_Hz	θ_F4
21	β_m-θ_AF4_T8	γ_m-α_CP1_AF4	β_m-β_T7	cfc_P7_28_Hz	θ_Fz

*Feature names listed should be self explanatory. The “ratio” features correspond to the log-ratio ones between the video and baseline periods; “AI” corresponds to the asymmetry index between the indicated channels*.

Table 5, in turn, reports the balanced accuracy results achieved with the individual features sets and the same dimensionality, as well as with the feature- and decision-level fusion strategies. All obtained results were significantly higher (*p* < 0.05) than those achieved with a random voting classifier (Koelstra et al., 2012). The column labeled “%” indicates the relative improvement in balanced accuracy, in percentage, relative to the SF baseline set. As can be seen, all proposed AMF features outperform the benchmark, by as much as 4.4, 5.6, 5.6, and 1.9% for valence, arousal, dominance, and liking, respectively. The PAC features also show advantages over the benchmark, particularly for the valence dimension, in which a 9.7% gain was observed. Feature fusion, in turn, showed to be useful mostly for arousal prediction, whereas decision-level fusion was useful for the liking dimension.

**Table 5:** Performance comparison of SVM classifiers for different feature sets and fusion strategies.

**Feature class**	**Fusion type**	**Valence**	**%**	**Arousal**	**%**	**Dominance**	**%**	**Liking**	**%**
AMI	–	0.604	4.4	0.583	5.6	0.564	4.1	0.626	1.1
AMC	–	0.594	2.7	0.563	1.9	0.569	5.0	0.630	1.9
AME	–	0.600	3.6	0.563	1.9	0.573	5.6	0.627	1.3
AMF	Feature-level	0.594	2.7	0.583	5.6	0.566	4.4	0.624	0.9
PAC	–	0.634	9.7	0.568	3.0	0.559	3.2	0.629	1.7
SF	–	0.578	–	0.552	–	0.542	–	0.619	–
AMF + SF + PAC	Feature-level	0.594	2.7	0.598	8.4	0.567	4.6	0.624	0.9
AMI + AMC + AME	Decision-level	0.594	2.8	0.563	1.9	0.567	4.6	0.625	1.0
AMF + PAC + SF	Decision-level	0.594	2.7	0.563	1.9	0.563	3.7	0.633	2.2

*All reported results were significantly higher than chance achieved with a random voting classifier (p < 0.05). Column labeled “%” indicates relative improvement, in percentage, over the SF baseline set*.

Moreover, for classifiers of varying dimensionality, maximum balanced accuracy values of 0.625 (AMI), 0.652 (AME), 0.659 (AMC) could be achieved for valence, dominance, liking, respectively, thus representing gains over the benchmark set of 8.1, 20.3, and 6.5%. For PAC features, gains could be seen only for the dominance dimension where a balanced accuracy of 0.592 could be seen, representing a gain over SF of 9.2%.

## 4. Discussion

### 4.1. Feature ranking

From Tables 1–4, it can be seen that with the exception of arousal, the number of SF features that passed the pre-screening test was roughly 20. For valence, roughly half those features corresponded to asymmetry index features, and across most emotional primitives, α, β and θ frequency bands showed to be the most relevant. These findings corroborate those widely reported in the literature (e.g., Davidson et al., 1979; Hagemann et al., 1999; Coan and Allen, 2004; Davidson, 2004).

Previous work on PAC, in turn, showed the coupling between EEG and GSR (computed via the ModI feature) to be relevant in emotion classification, particularly for arousal and valence (Kroupi et al., 2014). Interestingly, the CFC method of computing PANS-CNS phase-amplitude coupling was most often selected; for arousal 97% of the top features corresponded to CFC-type features. ModI features, in fact, were never selected as being a top candidate. PAC features showed to be particularly useful for valence estimation where 80% of the top features emanated from central brain regions (C3, CP1, FC1) and the attained balanced accuracy outperformed all other tested features. Such findings suggest that alternate PAC representations should be explored, especially within the scope of valence estimation.

Regarding the proposed AMF features, for arousal estimation, γ and β bands showed to be particularly useful, corresponding to roughly 86% of the top AMI features and 50% of the AMC and AME features. These findings are inline with results from Jenke et al. (2014). For valence, α interactions showed to be particularly useful, appearing in roughly 70% of the top AMI features. In particular α_m-θ interactions stood out, thus corroborating previous findings (Kensinger, 2004) which related these bands to states of internalized attention and positive emotional experience (Aftanas and Golocheikine, 2001). Such alpha/theta cross-frequency synchronization has also been previously related to memory usage (Chik, 2013). To corroborate this hypothesis, the correlation between the proposed features derived from the α_m-θ patterns and the subjective “familiarity” ratings reported by the participants was computed. The majority of the features showed to be significantly correlated (≥ 0.35, *p* < 0.05) with the familiarity rating, thus suggesting memory may have indeed played an effect on the elicited affective states.

Moreover, it was previously demonstrated that the power in the γ and β bands were also able to discriminate between liking and disliking judgements (Hadjidimitriou and Hadjileontiadis, 2012). By analyzing their amplitude modulation cross-frequency coupling via the proposed features, improved results were observed, thus showing the importance of EEG amplitude modulation coupling for affective state recognition. In fact, for the liking dimension 100% of the AMC features came from these two bands and this feature set resulted in the greatest improvement over the benchmark set (i.e., 1.9% increase). Moreover, β and α interactions were shown useful for dominance prediction in Liu and Sourina (2012). Here, 63% of the AMI features corresponded to those bands with several β_m-α features appearing at the top. Interestingly, for the AMC features, all top 19 features corresponded to β band interactions, with several coming from parietal regions, thus corroborating findings in Liu and Sourina (2012).

From the Tables, it can also be seen that the proposed normalization scheme over the baseline period was shown to be extremely important for the AME features, which unlike AMI and AMC, are energy-based features and not connectivity ones. For arousal, roughly 57% of the features corresponded to normalized features. For valence and liking they roughly corresponded to half of the top feature set. Normalization is important in order to remove participant-specific variability. Interestingly, only for the dominance dimension were normalized features seldom selected (20%) and it was for this emotional primitive that the AME features showed to be most useful. When analyzing the high/low threshold used per subject, it was observed that for the dominance dimension, the standard deviation of the optimal threshold across participants was lower at 0.65. For comparison purposes, the standard deviation for arousal (shown in Figure 3) was of 0.71. As such, since there was lower inter-subject variability for the dominance dimension, normalization was not as important. Overall, for the entire AMF set, channels that involved the frontal region provided several relevant features, thus confirming the importance of the frontal region for affective state recognition (Mikutta et al., 2012).

### 4.2. Classification and feature fusion

As shown in Table 5, all tested features and feature combinations resulted in balanced accuracy results significantly greater than chance. When all classifiers relied on the same input dimensionality and default parameters, the superiority of the proposed amplitude modulation features could be seen, particularly for the arousal, dominance and liking dimensions. In the case of equal dimensionality, fusion of AMF features did not result in any improvements over the individual amplitude modulation features, both for feature- and decision-level fusion. Notwithstanding, some improvement was seen when more features were explored. PAC features, in turn, were shown to be particularly useful for valence estimation. When PAC features were fused with benchmark and proposed AMF features, (i) feature-level fusion was shown to be particularly useful for arousal estimation, achieving results significantly better than the benchmark (*p* ≤ 0.05), and (ii) decision-level fusion was shown to be useful for liking prediction. Once varying input dimensionality was explored, the advantages of the proposed features over the benchmark became more evident, with gains as high as 8 and 20% being observed for the valence and dominance dimensions, respectively. Such results were significantly better than the benchmark (*p* ≤ 0.05).

### 4.3. Study limitations

This study has relied on the publicly available pre-processed DEAP database, which utilized a common average reference. Such referencing scheme could have introduced an artificial correspondence between nearby channels, thus potentially biasing the amplitude modulation and connectivity measures (Dezhong, 2001; Dezhong et al., 2005). By utilizing the multi-stage feature selection strategy, such biases were reduced, as feature redundancy was minimized and relevance was maximized. Moreover, from the relevant connections reported in the Tables, it can be seen that the majority of relevant connections are from electrodes that are sufficiently far apart, thus overcoming potential smearing contamination issues due to referencing. Moreover, as with many other machine learning problems, differences in data partitioning may lead to different top-selected features and, consequently, to varying performance results. This is particularly true for smaller datasets such as the one used herein. To test the sensitivity of data partitioning on feature selection, we randomly partitioned the 25% subset twice and explored the top selected features in each partition. For the AME features, for example, and the valence dimension, it was found that 13 of the top 23 features coincided for the two partitions. While this number is not very high, it is encouraging and future work should explore the use of boosting strategies and/or alternate data partitioning schemes to improve this.

## 5. Conclusions

In this work, experimental results with the publicly available DEAP database showed the EEG amplitude modulation based feature sets such as amplitude-amplitude cross-frequency modulation coupling features, as well as linear and nonlinear connection between multiple electrode pairs outperformed benchmark measures based on spectral power by as much as maximum 20% for dominance. Moreover, phase-amplitude coupling of EEG and GSR signals outperformed the benchmark by over 9% and when fused with the proposed amplitude modulation features, further gains in arousal and liking prediction were observed. Such findings suggest the importance of the proposed features for affective state recognition and signal the importance of EEG amplitude modulation for affective tagging of music video clips and content.

## Ethics statement

This study relied on publicly available data collected by others. Details about the database can be found at: Koelstra et al. (2012).

## Author contributions

AC performed data analysis and prepared the manuscript. RG assisted with data analysis and classification. SJ assisted with connectivity analysis and manuscript writing. TF contributed the study design, project supervision, and manuscript preparation. All authors read and approved the final manuscript.

### Conflict of interest statement

The authors declare that the research was conducted in the absence of any commercial or financial relationships that could be construed as a potential conflict of interest.
